# Neuroprotective mechanisms of erythropoietin in a rat stroke model

**DOI:** 10.1515/tnsci-2020-0008

**Published:** 2020-05-18

**Authors:** Martin Juenemann, Tobias Braun, Nadine Schleicher, Mesut Yeniguen, Patrick Schramm, Tibo Gerriets, Nouha Ritschel, Georg Bachmann, Martin Obert, Markus Schoenburg, Manfred Kaps, Marlene Tschernatsch

**Affiliations:** Department of Neurology, Justus-Liebig-University Giessen, Klinikstrasse 33, 35392, Giessen, Germany; Heart & Brain Research Group, Justus-Liebig-University Giessen and Kerckhoff Clinic, Benekestrasse 2-8, 61231, Bad Nauheim, Germany; Max-Planck-Institute for Heart and Lung Research, Ludwigstraße 43, 61231, Bad Nauheim, Germany; Department of Radiology, Kerckhoff Clinic, 61231, Bad Nauheim, Germany; Department of Radiology, Justus-Liebig-University Giessen, Klinikstrasse 33, 35392, Giessen, Germany; Department of Cardiac Surgery, Kerckhoff Clinic, 61231, Bad Nauheim, Germany; Department of Neurology, Gesundheitszentrum Wetterau, Chaumontplatz 1, 61231, Bad Nauheim, Germany

**Keywords:** neuroprotection, rat, recombinant human erythropoietin, transient focal cerebral ischemia, vascular disorders, craniectomy

## Abstract

**Objective:**

This study was designed to investigate the indirect neuroprotective properties of recombinant human erythropoietin (rhEPO) pretreatment in a rat model of transient middle cerebral artery occlusion (MCAO).

**Methods:**

One hundred and ten male Wistar rats were randomly assigned to four groups receiving either 5,000 IU/kg rhEPO intravenously or saline 15 minutes prior to MCAO and bilateral craniectomy or sham craniectomy. Bilateral craniectomy aimed at elimination of the space-consuming effect of postischemic edema. Diagnostic workup included neurological examination, assessment of infarct size and cerebral edema by magnetic resonance imaging, wet–dry technique, and quantification of hemispheric and local cerebral blood flow (CBF) by flat-panel volumetric computed tomography.

**Results:**

In the absence of craniectomy, EPO pretreatment led to a significant reduction in infarct volume (34.83 ± 9.84% vs. 25.28 ± 7.03%; *p* = 0.022) and midline shift (0.114 ± 0.023 cm vs. 0.083 ± 0.027 cm; *p* = 0.013). We observed a significant increase in regional CBF in cortical areas of the ischemic infarct (72.29 ± 24.00% vs. 105.53 ± 33.10%; *p* = 0.043) but not the whole hemispheres. Infarct size-independent parameters could not demonstrate a statistically significant reduction in cerebral edema with EPO treatment.

**Conclusions:**

Single-dose pretreatment with rhEPO 5,000 IU/kg significantly reduces ischemic lesion volume and increases local CBF in penumbral areas of ischemia 24 h after transient MCAO in rats. Data suggest indirect neuroprotection from edema and the resultant pressure-reducing and blood flow-increasing effects mediated by EPO.

## 1 Introduction

To date, many studies have been conducted on the identification, development, and evaluation of pharmacological neuroprotectants. In this context, experimental research has demonstrated that systemically administered recombinant human erythropoietin (rhEPO) partially crosses the blood–brain barrier (BBB) with a latency and is able to reduce neuronal damage in animal models of cerebrovascular, neuroinflammatory, and neurodegenerative diseases as well as traumatic central nervous system (CNS) injury [1,2,3,4]. Depending on the study design, preclinical studies on EPO in stroke models indicate an improvement in infarct size by up to 32% and neurobehavioral outcomes by almost 40% [5,6].

Erythropoietin production in the CNS seems to be triggered by hypoxia. Astrocytes, as well as oligodendrocytes, endothelial cells, neurons, and microglia, endogenously produce EPO. In principle, several types of EpoR receptors exist, including the homodimeric receptor (EpoR)_2_, a soluble as well as a heterodimeric receptor comprising a functional interaction of EpoR with the common β receptor (βcR, also known as CD131). The homodimeric EpoR has been detected on neural progenitor cells (NPCs), neurons, astrocytes, endothelial cells, and microglia [7,8,9]. Upregulation of EPO and EpoR in infarct and peri-infarct regions has been demonstrated in the course of focal cerebral ischemia/hypoxia [10]. The interaction of EPO and its receptor induces the phosphorylation of Janus kinase 2, which leads to the activation of phosphoinositide 3-kinase– serine–threonine kinase Akt and/or the signal transducer and activator of transcription 5 and/or the nuclear factor-κB pathway [11]. In this system, the EPO may exert neuroprotective effects via antiapoptotic mechanisms, stimulation of NPC proliferation and differentiation, neurogenesis, angiogenesis, and modification of inflammatory response and also induces erythropoiesis [7,8,12]. The heterodimeric EpoR/βcR receptor has been detected in various EPO-responsive tissues, including the cells of the CNS, such as microglia, and of the heart and kidney. It has been shown that this coexpression mediates the tissue-protective properties of EPO rather than erythropoietic effects [13].

Neuroprotection consists of prevention and opposition of pathological neuronal loss in diseases of the CNS [14]. Within cerebral ischemia, this loss can only partly be attributed to vessel occlusion. Moreover, perfusion deficits and the adjacent functional decline following cerebral vessel occlusion are consequences of the space-occupying effect of postischemic cerebral edema. Experimental data suggest that tissue swelling due to vasogenic edema during the hyperacute phase (<6 h) of stroke has considerable influence on temporospatial progression of the ischemic area by compromising microcirculation within critically perfused tissue at risk [15,16,17,18]. Therapeutic measures aiming at reducing cerebral edema and its space-occupying effect in the early stages of stroke may therefore induce an indirect “secondary” neuroprotection [16].

Most experimental studies focused on EPO treatment within the first hours following vessel occlusion [7], simulating the unpredictable situation clinicians face in the emergency department or in the stroke unit after sudden onset of a neurological deficit. However, with the advent of interventions in the cardio- and cerebrovascular systems – such as carotid endarterectomy and stenting, coronary artery bypass grafting, percutaneous coronary and cerebrovascular thrombectomy, angioplasty or coiling, and clipping of cerebral aneurysms – that carry an increased risk of stroke or require transient cerebral artery occlusion [19,20,21,22,23,24,25,26], anticipatory neuroprotection preceding a risk-related procedure demands greater attention [27]. In this context, experiments on a rodent model for transient middle cerebral artery occlusion (MCAO) suggest that beneficial effects of EPO treatment before ischemia onset can have a definite (if indirect) impact on the extent of ischemic edema and preservation of BBB function [27].

This study was designed to investigate secondary neuroprotective properties of rhEPO treatment preceding transient MCAO in a rodent stroke model. Dosage (5,000 IU/kg) and intravenous application were chosen according to the findings from the corresponding in vivo studies, considering the significantly low BBB permeability of this compound [1,7,28]. The multimodal approach included magnetic resonance imaging (MRI), flat-panel volumetric computed tomography (fpVCT), and quantification of brain water content (BWC) by the wet–dry technique. Elimination of the space-occupying effect of cerebral edema was achieved by bilateral craniectomy [29].

## 2 Methods

### 2.1 Animal preparation and surgical procedures

Male Wistar Unilever rats (HsdCpb:WU; Harlan Winkelmann, Germany) with a mean body weight of 310 g (±19.47 g) were used. Prior to surgery, each rat was administered 100 mg/kg metamizole (Novalgin^®^; Sanofi, Germany) orally. Anesthesia was established with 5% isoflurane delivered in air at 3.0 L/min and maintained during surgery via a facial mask with 2–3% isoflurane delivered in air at 0.5 L/min. The core body temperature was recorded with a feedback-controlled heating pad and kept at 37.0°C (±0.25°C) during surgery and imaging procedures.

In addition to considerable neurological deficits, rodents often exhibit pronounced cardiorespiratory instability after occlusion of the middle cerebral artery (MCA). Since this study was not intended to evaluate the craniectomy itself, but rather the effect of EPO under various pressure conditions, the craniectomy was performed before MCAO to avoid provoking an increased dropout rate through additional anesthesia and intervention with an already potentially unstable animal. Bilateral or sham craniectomies were performed after local anesthesia (2% lidocaine; Xylocaine®, AstraZeneca, Germany), as described previously [29]. The whole os parietale and the caudal parts of the os frontale were removed using a liquid-cooled trephine, while the dura mater was left intact.

Afterward, the animals were randomized to treatment with EPO or placebo and MCAO was performed in each rat as discussed previously [15]. In brief, the right common carotid artery was exposed and a silicone-coated nylon suture (4-0) was inserted. Then the occluder was advanced proximally until its tip reached the anterior cerebral artery (mean suture depth: 20 ± 2 mm) beyond the carotid bifurcation, thus blocking the blood flow to the right MCA. Reperfusion was established after 90 minutes by removing the suture. Metamizole was administered orally again 6 h after the first application and added to the tap water.


**Ethical approval:** The research related to animal use has been complied with all the institutional guidelines and the current German animal protection law. The experiments were approved by the regional committee for the care and use of animals (Regierungspraesidium Darmstadt; Az.B2/170).

### 2.2 Experimental setup

One hundred and ten rats were randomly assigned to four groups: (i) placebo + craniectomy, (ii) EPO + craniectomy, (iii) placebo–craniectomy, and (iv) EPO–craniectomy.

Craniectomy was performed in 56 animals (+craniectomy); the bone skull of 54 rats was thinned but not completely removed (–craniectomy). Thereafter, all 110 rats were randomly subjected to the treatment groups (EPO vs. placebo): 15 minutes prior to MCAO, each animal was administered 5,000 IU/kg EPO (NeoRecormon®; Roche, Germany) in 2 ml isotonic saline (EPO) or only 2 ml isotonic saline (placebo) via coccygeal venous catheter. Afterward, MCAO was performed by a surgeon blinded to the group assignment. Functional testing took place at baseline and 24 h after MCAO. Then ten rats of each group were subjected to MRI to detect ischemic lesion volume, vascular edema, and midline shift (MLS) and to postmortem quantification of BWC by the wet–dry technique. The remaining animals of each group underwent quantification of cerebral blood flow (CBF) via fpVCT. Functional assessment, radiological imaging, and evaluation, as well as wet–dry analysis, were performed by experienced investigators blinded to the group assignment.

### 2.3 Functional testing

Motor functions were assessed using the Rotarod test at baseline and 24 h after MCAO. The wheel was continuously accelerated from 0 to 30 rpm within 1 minute. The maximum speed tolerated by the rats was documented and the difference was calculated as Rotarod performance before and after MCAO [30].

### 2.4 MRI

After functional testing, the MRI scanning was performed under anesthesia with a tomography (Bruker PharmaScan 7.0 T, 16 cm), which operates at 300.51 MHz (1H-imaging) and is equipped with a 300 mT/m self-shielding gradient system. The animal’s respiratory rate was monitored noninvasively and maintained between 60 and 80/min by regulation of the isoflurane concentration.

The linear polarized volume resonator (diameter 60 mm) was tuned and matched manually, and localized images were acquired using a spin-echo sequence. Rapid acquisition with relaxation enhancement sequences (20 contiguous slices of 1 mm thickness, repetition time [TR] = 2500 ms, and echo time [TE] = 41.8 ms) were used to verify symmetric positioning and were repeated after correction of the possible necessary slice angulation [18].

### 2.5 T2-imaging

To map the vascular edema (T2-relaxation time [T2RT]) [15] and the lesion and hemispheric volumes, we used a Carr–Purcell–Meiboom–Gill spin echo imaging sequence, acquiring eight contiguous coronal slices (slice thickness = 2 mm, gap = 0 mm, field-of-view (FOV) = 37 × 37 mm, matrix size = 512 × 256, TR = 3833.5 ms, TE [12 echos, ΔTE = 18 ms] = 18–216 ms, number of excitations (NEX) = 1, and acquisition time (AT) = 12 min 7 s).

### 2.6 T2*-imaging

To exclude animals with possible hemorrhages, 16 contiguous coronal slices were acquired using an SNAP-T2*-imaging sequence (slice thickness = 1 mm, gap = 0 mm, FOV = 37 × 37 mm, matrix size 256 × 256, TR = 43.4 ms, TE = 7.0 ms, and AT = 12 min 7 s).

### 2.7 MRI data evaluation

#### 2.7.1 Ischemic lesion volume

The mean ischemic lesion volume was determined by performing computer-aided planimetric assessment of the lesion volume (LV) and the hemispheric volumes of the T2-weighted images (ipsilateral: HV_i_; contralateral: HV_c_) (ImageJ v1.46; National Institutes of Health, Bethesda, USA). The edema-corrected lesion volume (%HLV_ec_) was calculated by the following equation [18]:

(1) \% {\text{HLV}}_{\text{ec}}=(({\text{HV}}_{\text{c}}-{\text{HV}}_{\text{i}}+\text{LV}}/{\text{HV}}_{\text{c}})\times 100

#### 2.7.2 MLS quantification

The MLS quantification was performed using high-resolution T2-weighted images. The position of the third ventricle could be clearly determined in all rats. The distance between the middle of the third ventricle and the outer border of both hemispheres (distance from ipsilateral border to third ventricle: *A* and distance from contralateral border to third ventricle: *B*) was measured [17] and MLS was calculated by the following equation:

(2) \text{MLS}=\text{(}A-B\text{)/}2

#### 2.7.3 T2RT

For quantification of the T2RT, we used Bruker’s implemented image processing tool. On the six contiguous coronal slices, regions of interest (ROIs) were set in the center of the ischemic lesions in the cortex and subcortex and on the corresponding position of the contralateral hemisphere, and the side-to-side differences of the T2RT were calculated.

### 2.8 Postmortem analysis: quantification of BWC by the wet–dry technique

After MRI, the animals were deeply anesthetized and decapitated. The brains were removed and separated into the ipsi- and contralateral hemispheres. The wet weight of each hemisphere was measured, then the tissue was dried to a constant weight at 50°C and weighed again (dry weight). The absolute BWC (%H_2_O) was calculated as follows [18]:

(3) \% {\text{H}}_{2}\hspace{-.25em}\text{O}\hspace{.50em}=\hspace{.25em}([\text{wet}\hspace{.25em}\text{weight -- dry}\hspace{.25em}\text{weight}]\text{/wet}\hspace{.25em}\text{weight}\times 100)

Equation (4) was used to calculate the increase in BWC in the ipsilateral hemisphere compared to the unaffected contralateral hemisphere (%ΔH_2_O) [18]:

(4) \% {\text{\Delta{H}}}_{2}\hspace{-.25em}\text{O}\hspace{.50em}= \% \hspace{.0em}{\text{H}}_{2}{\text{O}}_{\text{ipsilateral}}- \% {\text{H}}_{2}{\text{O}}_{\text{contralateral}}

### 2.9 fpVCT

The CBF was quantified after the 24-h clinical testing with an fpVCT, which was developed by GE Healthcare, London, Ontario, Canada. The system is described in detail in the study by Obert et al. (2010) [31]. Preparation and anesthesia of the rats, image acquisition, reconstruction, and analysis followed a previously published protocol [32].

### 2.10 Placing the ROIs, infarct core, and hemisphere

Since the infarcted brain regions cannot be properly displayed on perfusion slices, the corresponding 2,3,5-triphenyltetrazolium chloride (TTC)-stained slices were used to identify the extent and location of ischemic areas. After VCT investigation, the animals were deeply anesthetized using isoflurane and euthanized by decapitation; the brains were removed and sectioned coronally into six slices (thickness: 2 mm each), incubated in a 2% solution of TTC at 37°C, fixed by immersion in 10% buffered formalin solution, and scanned with a computer scanner (ScanJet 3400C; Hewlett Packard; resolution 600 × 600 dpi). The unstained areas of the fixed brain slices were defined as the ischemic infarction.

For ipsi- and contralateral sides, flexibly created freehand ROIs included cortical and subcortical regions of the infarct core as well as the whole hemisphere. The CBF (ml/100 g/min) was acquired as mean for each side or the corresponding region and in each animal, thus permitting comparison of data between infarct hemisphere and non-infarct hemisphere. Differences in CBF were calculated by the following equation:

(5) \hspace{-8em}\text{\%CBF}\hspace{.25em}\text{difference}\hspace{-7em}=(\text{mean}\hspace{.25em}\text{CBF}\hspace{.25em}\text{ipsilateral/mean}\hspace{.25em}\text{CBF}\hspace{.25em}\text{contralateral})\hspace{6em}\hspace{-6em}\times \text{100}

### 2.11 Statistical analysis

The Shapiro–Wilk test was used to test for normal distribution of parametric data. Homogeneity of variance was tested by the Levene test. Erythropoietin treatment and placebo groups were compared separately for craniectomy and sham craniectomy by unpaired Student’s *t* test or, for data not passing the normality test, the nonparametric Mann–Whitney *U* test. Data are presented as mean ± standard deviation. The level of probability *p* < 0.05 was regarded as significant (SPSS v21; IBM, Germany).

## 3 Results

Twenty-six animals had to be excluded from this study: for seven animals, technical problems occurred during contrast agent infusion, and the imaging of another four rats was hampered due to motion artifacts. Seven animals suffered cerebral hemorrhage, six animals died during craniectomy, and two rats showed no ischemic infarction. The remaining 84 animals completed the study protocol.

Pre-MCAO Rotarod performance, body weight, and body temperature did not differ significantly between the groups.

### 3.1 Neurological impairment

The results in Rotarod test performance pre- vs. 24 h post-MCAO did not differ significantly between the craniectomy (5.71 ± 11.14 rpm vs. 6.29 ± 9.95 rpm; *p* = 0.837) and sham craniectomy groups (5.43 ± 11.21 rpm vs. 6.00 ± 7.10 rpm; *p* = 0.786).

### 3.2 Infarct size

**Figure 1:**
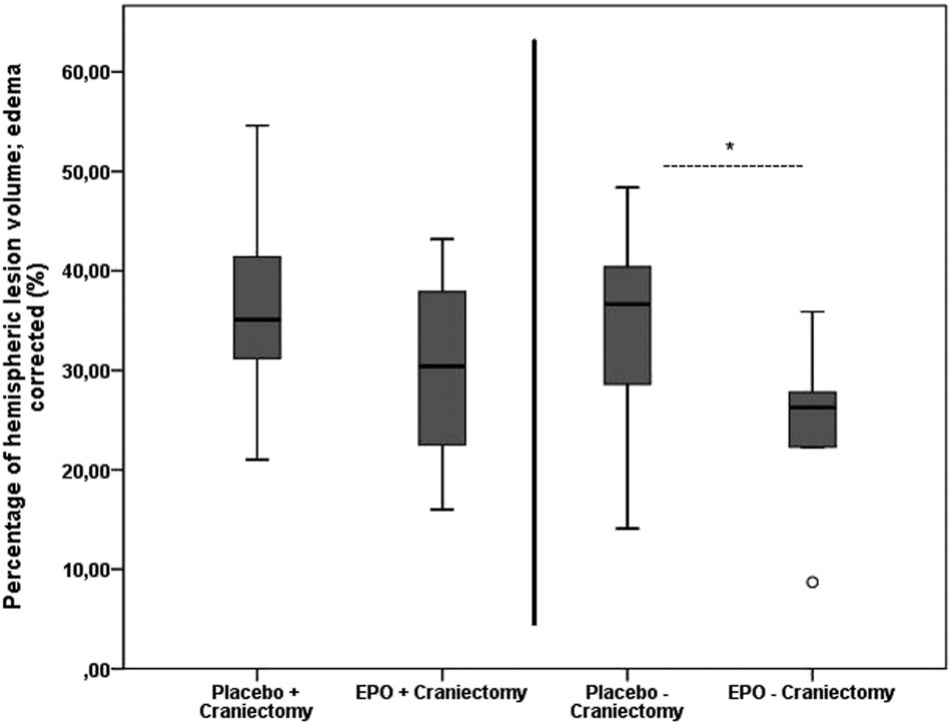
Mean ischemic lesion volume as determined on MRI, expressed in percentage of hemispheric volume (%HLV_ec_). Significantly reduced mean ischemic lesion volume for EPO-treated animals compared to the placebo group without craniectomy (*p = 0.022; *t*-test). No significant difference could be detected between craniectomy groups. Outliers are marked with a circle (out values) or a star (far out values).

Measurement by MRI of infarct sizes corrected for the space-occupying effect of brain edema revealed no difference (*t*(18) = 1.391; *p* = 0.181, *d* = 0.62) between craniectomy rats receiving placebo (36.29 ± 10.21%) vs. EPO (30.34 ± 8.86%). The mean ischemic lesion volume after 24 h was significantly smaller (*t*(18) = 2.497; *p* = 0.022, *d* = 1.12) in EPO-treated animals without craniectomy (25.28 ± 7.03%) when compared to placebo animals without craniectomy (34.83 ± 9.84%) (Figure 1).

### 3.3 Brain edema

**Figure 2:**
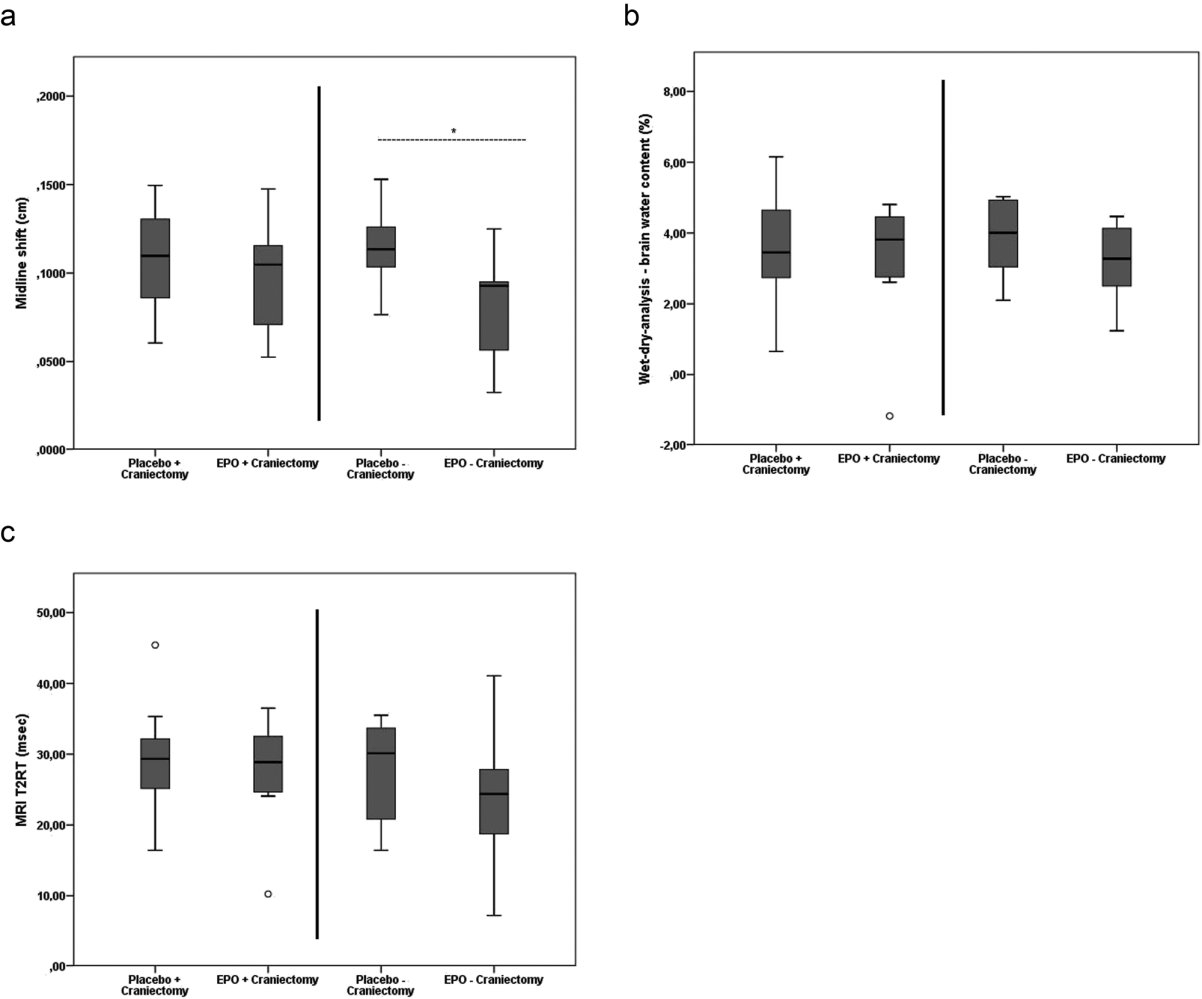
MLS (a), BWC (b), and T2RT (c). (a) MLS in the groups without craniectomy was significantly smaller in animals treated with EPO when compared to the placebo animals (*p = 0.013). No significant differences were observed within the craniectomy groups. (b) BWC and (c) T2RT displayed no significant differences between the groups. Outliers are marked with a circle (out values) or a star (far out values).

The MLS of the animals treated with EPO (0.083 ± 0.027 cm), which were not craniectomized, was significantly reduced (*t*(18) = 2.768; *p* = 0.013; *d* = 1.24) when compared to the noncraniectomized placebo animals (0.114 ± 0.023 cm). This could not be observed in the two craniectomy groups (placebo: 0.109 ± 0.029 cm vs. EPO: 0.100 ± 0.031 cm, respectively; *t*(18) = 0.613; *p* = 0.548; *d* = 0.30) (Figure 2a).

In the EPO- and placebo-treated craniectomy groups, BWC (placebo: 3.33 ± 1.75% vs. EPO: 3.48 ± 1.61%, respectively; *z* = 1.71; *p* = 0.912; *d* = 0.09) and T2RT (placebo: 29.50 ± 7.79 ms vs. EPO: 27.57 ± 7.39 ms, respectively; *t*(18) = 0.569; *p* = 0.576; *d* = 0.25) showed no significant difference. A similar result could be obtained from the analyses of BWC (placebo: 3.87 ± 1.02% vs. EPO: 3.19 ± 1.11%; *z* = −1.21; *p* = 0.247; *d* = 0.64) and T2RT (placebo: 28.25 ± 6.65 ms vs. EPO: 23.54 ± 9.14 ms; *t*(18) = 1.318; *p* = 0.204; *d* = 0.59) in noncraniectomy groups III + IV (Figure 2b and c).

### 3.4 CBF

CBF was acquired within cortical and subcortical regions of the infarct core as well as the whole hemisphere and expressed as a ratio between the ipsilateral and the contralateral sides.

**Figure 3:**
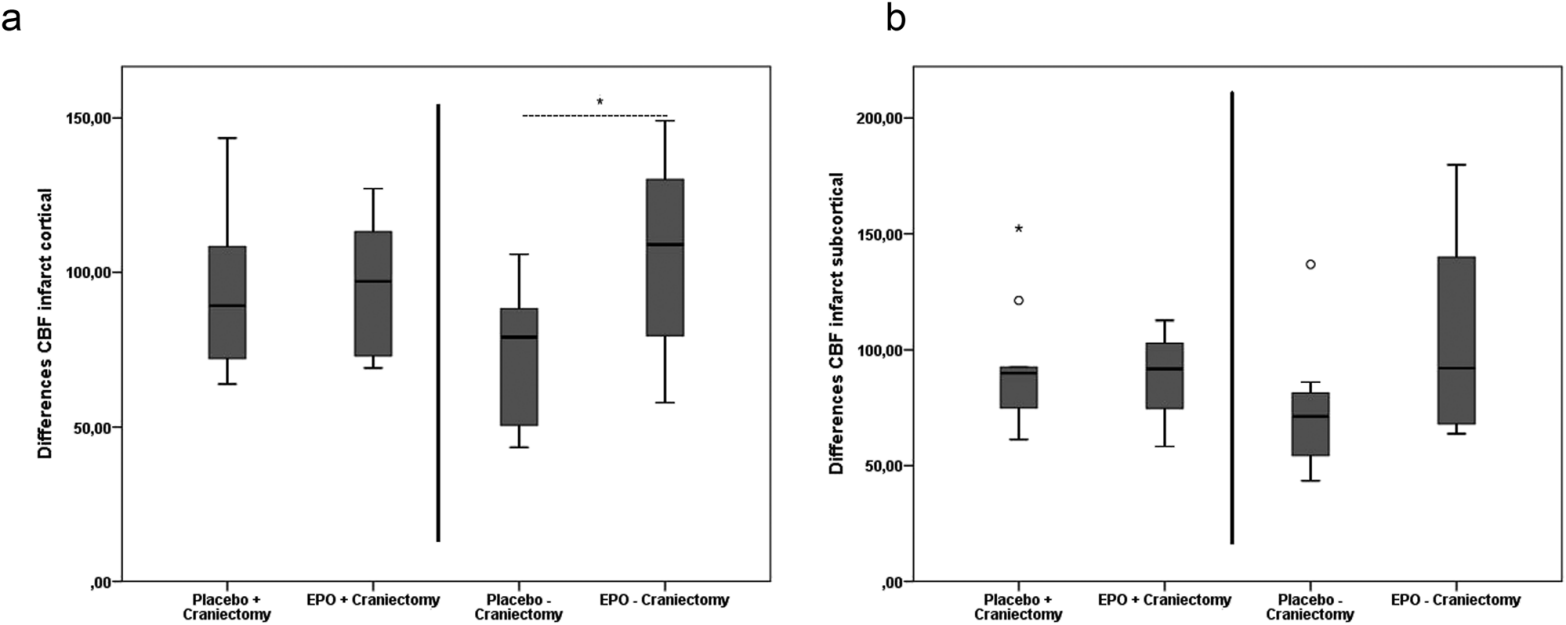
CBF in the infarct core. There was a significant CBF increase with EPO treatment in the cortical regions of the infarct core when compared to placebo treatment in the absence of craniectomy (*p = 0.043 *u*-test) (a). No significant CBF changes could be observed in the cortical regions of the infarct core when craniectomy was performed (a) or in subcortical regions (b). Outliers are marked with a circle (out values) or a star (far out values).

In the absence of craniectomy (groups III + IV), the EPO-treated animals showed a significant increase in CBF in cortical regions of the infract core when compared to the placebo-treated animals (placebo: 72.29 ± 24.00% vs. EPO: 105.53 ± 33.10%, respectively; *t*(18) = −2.245; *p* = 0.043; *d* = 1.00) (Figure 3a). In the subcortical regions (placebo: 74.29 ± 29.04 vs. EPO: 103.38 ± 39.54%, respectively; *t*(18) = −1.799; *p* = 0.091; *d* = 0.85) and the total infarct core (placebo: 76.58 ± 28.03 vs. EPO: 104.48 ± 33.49%, respectively; *t*(18) = −1.975; *p* = 0.065; *d* = 0.90), tendencies did not reach statistical significance. No significant difference in CBF was observed in the cortical (placebo: 94.40 ± 28.62% vs. EPO: 95.12 ± 23.79%, respectively; *t*(18) = −0.05; *p* = 0.961; *d* = 0.03) or subcortical region (placebo: 88.94 ± 19.00% vs. EPO: 92.74 ± 26.73%, respectively; *t*(18) = 0.378; *p* = 0.709; *d* = 0.16) or the whole infarct core within the craniectomy groups (placebo: 90.89 ± 20.39% vs. EPO: 96.67 ± 28.19%, respectively; *t*(18) = 0.542; *p* = 0.594; *d* = 0.23) (groups I + II, Figure 3).

**Figure 4:**
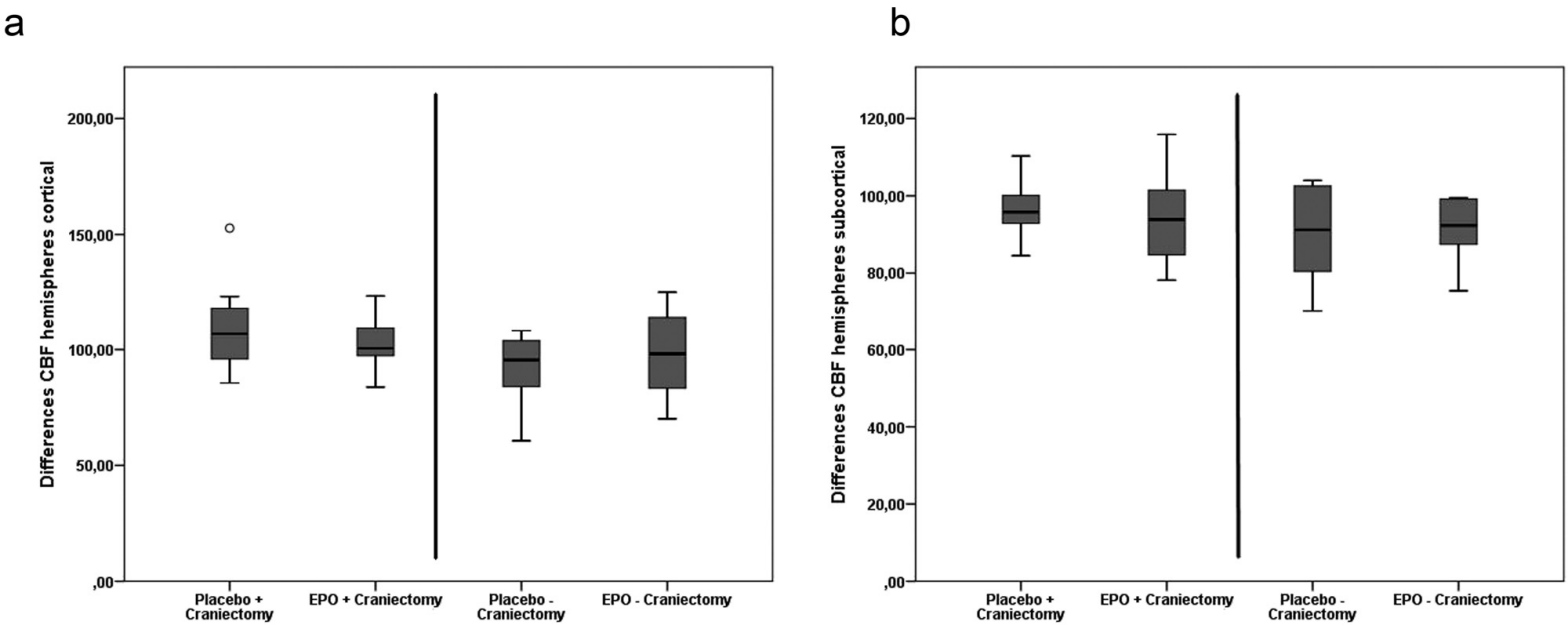
CBF in the hemisphere. The CBF measurement of the whole hemispheres, i.e., cortical (a) and subcortical (b) regions, revealed no significant effects of EPO treatment regardless of craniectomy. Outliers are marked with a circle (out values).

Between the craniectomy groups, investigation of hemispherical blood flow in the cortical (placebo: 108.59 ± 19.46% vs. EPO: 103.75 ± 11.68%, respectively; *t*(18) = 0.706; *p* = 0.488; *d* = 0.30) and subcortical regions (placebo: 96.94 ± 7.15% vs. EPO: 94.11 ± 11.30%, respectively; *t*(18) = 0.699; *p* = 0.492; *d* = 0.29) and total hemisphere (placebo: 103.70 ± 15.81% vs. EPO: 100.13 ± 10.96%, respectively; *t*(18) = 0.616; *p* = 0.545; *d* = 0.26) revealed no significant effects of EPO treatment. Similar results were shown in the comparison of CBF between groups III + IV without craniectomy in the cortical (placebo: 91.25 ± 16.45% vs. EPO: 99.12 ± 18.04%, respectively; *t*(18) = −1.020; *p* = 0.312; *d* = 0.46) and subcortical regions (placebo: 90.02 ± 11.54% vs. EPO: 90.43 ± 9.18%, respectively; *t*(18) = −0.087; *p* = 0.931; *d* = 0.04) and total hemisphere (placebo: 91.26 ± 11.86% vs. EPO: 94.36 ± 10.90%, respectively; *t*(18) = −0.609; *p* = 0.550; *d* = 0.27) (Figure 4).

## 5 Discussion

Investigations on rodent stroke models [16,17,18,33] indicate that the development of vasogenic brain edema within the hyperacute phase of stroke (<6 h) may exert a significant, possibly underestimated, influence on the progression of ischemic area, as swelling of ischemic tissue within the fixed cranial volume can lead to impairment of microcirculation in the critically hypoperfused penumbral area. Hence, collateral damage caused by the space-occupying effect of a large MCA territory stroke accounts for up to 50% of ischemic lesion formation [16]. Therapeutic measures aiming to reduce cerebral edema and its resulting space-occupying effect in the early stages of stroke may operate as indirect or “secondary” neuroprotectants [16,29]. Investigations on the effects of systemically administered rhEPO prior to transient MCAO in rodents suggest that neuroprotection results more from the mitigation of brain edema than from direct antiapoptotic effects on neurons [27]. We hypothesized that EPO administered prior to transient MCAO exerts its neuroprotective properties in the early phase of stroke primarily via secondary neuroprotection by reduction of cerebral edema.

Craniectomy has been shown to save the lives of patients with large space-occupying territorial strokes in severe danger of cerebral herniation and death and was proven in large clinical trials to reduce mortality significantly, from 71 to 22% [34,35]. Experimental studies on the effect of craniectomy in a rodent model of MCAO report a significant reduction of infarct size, mainly attributed to the release of mechanical compression [36,37]. To approach the distinction of primary from secondary neuroprotection in our study, elimination of increased intracranial pressure due to the space-occupying ischemia was achieved by bilateral craniectomy prior to transient MCAO [16,29]. Thus, the preponderance of edema reduction via EPO was expected to lead to pronounced group differences regarding infarct size and edema volume, which are dependent on integrity of the skull.

We observed that rhEPO treatment before transient MCAO reduced edema-corrected infarction size by approximately 10%. Data on experiments with a comparable setting are limited; two previous studies on rats reported no significant effects on infarct volume for EPO pretreatment. In contrast, a study on mice showed infarct reduction up to 47% [27,38,39]. Interestingly, in the present investigation, a significant reduction compared to placebo treatment could only be observed if the skull was left intact; an approximation of infarct sizes could be quantified with craniectomy.

Experimental research on rodent stroke models provides robust evidence for the antiedematic effects of EPO, which has particularly been attributed to a preserved barrier function of the BBB [40,41,42,43,44,45]. An investigation on the markers of BBB integrity – such as occludin, alpha-, and beta-catenin – demonstrated that EPO treatment before and 3 days after focal cerebral ischemia can stabilize the BBB, reduce its permeability, and thereby control cerebral inflammation and edema [43]. Impermeability of the BBB mainly depends on intact endothelial cells and tight junctions, which are subjected to substantial oxidative stress by generation of reactive oxygen species and lipid peroxidation during the phase of reperfusion after transient ischemia [46,47]. Under this condition, EPO seems to stimulate endothelial nitric oxide production and has the ability to prevent reperfusion-mediated injury to the BBB [48]. Due to the fact that lesion volume is proportional to hemispheric BWC, the volume of infarcted tissue can bias methods for quantification of BWC that include whole hemispheres, such as the wet–dry technique and determination of MLS; cerebral edema in the present investigation was therefore assessed on MRI using T2RT measurements in ROIs, since this method was shown to be largely independent of lesion size [18]. We could demonstrate a significant reduction in MLS for EPO pretreatment only in the absence of bilateral craniectomy. In this group, MRI T2RT presented a trend toward the lowest mean values for the treatment group with intact skull but missed statistical significance. Nevertheless, these data seem to suggest that neuroprotection of EPO pretreatment in transient MCAO implies a strong antiedematic effect.

We used fpVCT for noninvasive dynamic imaging of cerebral perfusion after temporary MCAO in the cortical and subcortical regions of the infarct and the whole hemisphere [32]. Without craniectomy, the EPO pretreatment led to a significant increase in CBF in the cortical regions of the ischemic tissue. However, in subcortical areas of the infarct and the whole hemispheres, no significant alterations of CBF could be objectified. Xiong et al. described EPO neuroprotection after traumatic brain injury even in EpoR null mice and attributed this effect particularly to vascular protection [49]. Li et al. investigated angiogenesis in mice that received rhEPO 30 minutes before and once daily after ischemic stroke and observed enhanced angiogenic activity between days 7 and 21; on day 14, the CBF reached preischemia initial values [50]. Furthermore, in a rabbit model for subarachnoid hemorrhage, intravenously administered rhEPO led to a significantly increased CBF between days 2 and 16 [51]. In addition to these observations of EPO’s time-sensitive effects, Shafi et al. used isolated rat MCA to demonstrate that luminal-applied EPO can directly dilate arteries and that 24-h pretreatment with EPO potentiates this effect [52]; after this single-dose pretreatment with EPO and transient MCAO, we only observed a significant increase in CBF in the defined cortical regions of the infarct and in the absence of craniectomy. If compression on the brain, microvasculature and presumable pial and venous vessels is released by craniectomy, the CBF in EPO- and placebo-treated rats is equal. This seems to display a local effect for the defined area of the ischemia, as no differences in CBF could be observed for total hemispheres regardless of the EPO treatment or craniectomy. A focal improvement in CBF in the cortical regions of the ischemic area may indicate a more efficient collateralization with EPO, either via its antiedemic and pressure-reducing mode of action or due to its direct vasodilatative effects. Improved collateralization in turn supports the recovery of critically perfused penumbral areas reducing the infarct core, which has been shown by a significant reduction in ischemic lesion volume. In line with the aforementioned data on infarct size and edema reduction, the latter could only be objectified in the absence of craniectomy, i.e., a situation in which pressure variations are supposed to be the most pronounced.

The results of this study have to be interpreted with caution, as surrogate parameters for a secondary neuroprotective mechanism of action were considered. These can be regarded as hypothesis generating but must subsequently be confirmed in the corresponding mechanistic studies to objectively distinguish a direct or indirect mechanism of action.

In this study, rhEPO was administered – a compound that, because of its low BBB permeability, must be applied in comparatively high intravenous doses, prompting several dose-dependent side effects such as increased hematocrit and hypertension as well as procoagulatory and prothrombotic effects on microcirculation. These side effects seem to be primarily due to the erythropoietic mode of action of the EPO derivate, and it is conceivable in principle that they limit the extent of neuroprotection in the context of acute cerebrovascular diseases. Therefore, efforts have been made in the past to support EPO-mediated cytoprotection without affecting the hematopoietic system. In this respect, it could be shown that, putatively due to an altered receptor interaction, carbamylated EPO and mutants such as EPO-S100E or EPO-R103E act neuroprotectively but lack erythropoietic activity with a drastically reduced (EPOR)_2_ affinity. In addition, the fusion protein EPO-Tat possesses a significantly enhanced BBB permeability and thus enables the use of lower effective doses [53,54]. It therefore remains to be discussed whether the use of another EPO derivative yielded different, clearer results.

Another aspect of pharmacokinetics appears to be of particular interest in connection with the application of EPO prior to MCAO. In a rodent model of traumatic brain injury, it was shown not only that EPO must be administered in high doses when applied peripherally and that intravenous is superior to intraperitoneal administration but also that rhEPO crosses the BBB with a delay of approximately 4 h and appears to develop its biological effect after around 8 h [1]. Moreover, the half-life of rhEPO after single injection was reported to be between 25.6 h and 35.5 h [2]. If this time frame of pharmacokinetics and the edema dynamics after cerebral infarction with the onset immediately after ischemia are taken into account, an even earlier time of application of EPO could possibly have led to a more pronounced neuroprotective effect. Thus, the single administration of EPO immediately after the onset of ischemia, which is more similar to the clinical situation in stroke, is not expected to produce significantly different results. As described in the introduction, the timing was chosen against the background of anticipatory neuroprotection in cerebrovascular interventions, for example, where the onset of damage is known; a transfer of the results to acute stroke therapy is only possible with difficulty.

With respect to the effect sizes of infarct volumes and perfusion parameters, although the number of animals per group seems to be sufficient, it remains debatable whether larger groups for investigations on brain edema would have led to significantly different results.

Clinical testing did not point out a statistically significant functional improvement, which might indicate limited sensitivity of the clinical tests in general or with regard to the chronological parameters and points in time selected in this study. Furthermore, the study design does not allow quantification of possible long-term improvement. In principle, the use of healthy animals, controlled laboratory conditions, and application of anesthesia can hamper the assignability of findings from bench to bedside, which has to be considered when findings are interpreted.

## 6 Conclusion

Interventions for predictable stroke risk substantiate discussion on anticipatory neuroprotection preceding the risk-related procedure and intended for prevention of neuronal loss. This study demonstrates that a single dose of rhEPO 5,000 IU/kg given prior to transient MCAO in rats significantly reduces ischemic lesion volume, decreases MLS, and increases local CBF in the cortical regions of ischemia after 24 h. Data may suggest an interaction between edema and pressure reducing as well as blood flow-increasing effects mediated by EPO.

## References

[1] Lieutaud T, Andrews PJD, Rhodes JKJ, Williamson R. Characterization of the pharmacokinetics of human recombinant erythropoietin in blood and brain when administered immediately after lateral fluid percussion brain injury and its pharmacodynamic effects on IL-1beta and MIP-2 in rats. J Neurotrauma. 2008;25:1179–85. 10.1089/neu.2008.0591 18842103

[2] Xenocostas A, Cheung WK, Farrell F, Zakszewski C, Kelley M, Lutynski A, et al. The pharmacokinetics of erythropoietin in the cerebrospinal fluid after intravenous administration of recombinant human erythropoietin. Eur J Clin Pharmacol. 2005;61:189–95. 10.1007/s00228-005-0896-7 15776276

[3] Sargin D, Friedrichs H, El-Kordi A, Ehrenreich H. Erythropoietin as neuroprotective and neuroregenerative treatment strategy: comprehensive overview of 12 years of preclinical and clinical research. Best Pract Res Clin Anaesthesiol. 2010;24:573–94. 10.1016/j.bpa.2010.10.005 21619868

[4] Brines ML, Ghezzi P, Keenan S, Agnello D, de Lanerolle NC, Cerami C, et al. Erythropoietin crosses the blood-brain barrier to protect against experimental brain injury. Proc Natl Acad Sci USA. 2000;97:10526–31. 10.1073/pnas.97.19.10526 PMC27058 10984541

[5] Minnerup J, Heidrich J, Rogalewski A, Schäbitz W-R, Wellmann J. The efficacy of erythropoietin and its analogues in animal stroke models: a meta-analysis. Stroke. 2009;40:3113–20. 10.1161/STROKEAHA.109.555789 19542052

[6] Jerndal M, Forsberg K, Sena ES, Macleod MR, O’Collins VE, Linden T, et al. A systematic review and meta-analysis of erythropoietin in experimental stroke. J Cereb Blood Flow Metab. 2010;30:961–8. 10.1038/jcbfm.2009.267 PMC2949185 20040929

[7] van der Kooij MA, Groenendaal F, Kavelaars A, Heijnen CJ, van Bel F. Neuroprotective properties and mechanisms of erythropoietin in in vitro and in vivo experimental models for hypoxia/ischemia. Brain Res Rev. 2008;59:22–33. 10.1016/j.brainresrev.2008.04.007 18514916

[8] Noguchi CT, Asavaritikrai P, Teng R, Jia Y. Role of erythropoietin in the brain. Crit Rev Oncol Hematol. 2007;64:159–71. 10.1016/j.critrevonc.2007.03.001 PMC2083122 17482474

[9] Minnerup J, Wersching H, Schäbitz W-R. EPO for stroke therapy - Is there a future for further clinical development? Exp Transl Stroke Med. 2010;2:10. 10.1186/2040-7378-2-10 PMC2880017 20459870

[10] Sirén AL, Knerlich F, Poser W, Gleiter CH, Brück W, Ehrenreich H. Erythropoietin and erythropoietin receptor in human ischemic/hypoxic brain. Acta Neuropathol. 2001;101:271–6. 10.1007/s004010000297 11307627

[11] Sola A, Wen T-C, Hamrick SEG, Ferriero DM. Potential for protection and repair following injury to the developing brain: a role for erythropoietin? Pediatr Res. 2005;57:110R–7R. 10.1203/01.PDR.0000159571.50758.39 15817504

[12] Minnerup J, Schäbitz W-R. Multifunctional actions of approved and candidate stroke drugs. Neurotherapeutics. 2009;6:43–52. 10.1016/j.nurt.2008.10.032 PMC5084255 19110198

[13] Brines M, Grasso G, Fiordaliso F, Sfacteria A, Ghezzi P, Fratelli M, et al. Erythropoietin mediates tissue protection through an erythropoietin and common beta-subunit heteroreceptor. Proc Natl Acad Sci USA. 2004;101:14907–12. 10.1073/pnas.0406491101 PMC522054 15456912

[14] Sirén AL, Ehrenreich H. Erythropoietin – a novel concept for neuroprotection. Eur Arch Psychiatry Clin Neurosci. 2001;251:179–84. 10.1007/s004060170038 11697582

[15] Gerriets T, Stolz E, Walberer M, Müller C, Rottger C, Kluge A, et al. Complications and pitfalls in rat stroke models for middle cerebral artery occlusion: a comparison between the suture and the macrosphere model using magnetic resonance angiography. Stroke. 2004;35:2372–7. 10.1161/01.STR.0000142134.37512.a7 15345802

[16] Walberer M, Ritschel N, Nedelmann M, Volk K, Mueller C, Tschernatsch M, et al. Aggravation of infarct formation by brain swelling in a large territorial stroke: a target for neuroprotection? J Neurosurg. 2008;109:287–93. 10.3171/JNS/2008/109/8/0287 18671642

[17] Walberer M, Blaes F, Stolz E, Müller C, Schoenburg M, Tschernatsch M, et al. Midline-shift corresponds to the amount of brain edema early after hemispheric stroke – an MRI study in rats. J Neurosurg Anesthesiol. 2007;19:105–10. 10.1097/ANA.0b013e31802c7e33 17413996

[18] Gerriets T, Stolz E, Walberer M, Müller C, Kluge A, Bachmann A, et al. Noninvasive quantification of brain edema and the space-occupying effect in rat stroke models using magnetic resonance imaging. Stroke. 2004;35:566–71. 10.1161/01.STR.0000113692.38574.57 14739415

[19] Daou B, Chalouhi N, Starke RM, Barros G, Ya’qoub L, Do J, et al. Clipping of previously coiled cerebral aneurysms: efficacy, safety, and predictors in a cohort of 111 patients. J Neurosurg. 2016;125:1337–43. 10.3171/2015.10.JNS151544 26894462

[20] Ha S-K, Lim D-J, Seok B-G, Kim S-H, Park J-Y, Chung Y-G. Risk of stroke with temporary arterial occlusion in patients undergoing craniotomy for cerebral aneurysm. J Korean Neurosurg Soc. 2009;46:31–7. 10.3340/jkns.2009.46.1.31 PMC2729821 19707491

[21] Goyal M, Menon BK, van Zwam WH, Dippel DWJ, Mitchell PJ, Demchuk AM, et al. Endovascular thrombectomy after large-vessel ischaemic stroke: a meta-analysis of individual patient data from five randomised trials. Lancet. 2016;387:1723–31. 10.1016/S0140-6736(16)00163-X 26898852

[22] Gill HL, Siracuse JJ, Parrack I-K, Huang ZS, Meltzer AJ. Complications of the endovascular management of acute ischemic stroke. Vasc Health Risk Manag. 2014;10:675–81. 10.2147/VHRM.S44349 PMC4259256 25506222

[23] Palmerini T, Biondi-Zoccai G, Della Riva D, Mariani A, Savini C, Di Eusanio M, et al. Risk of stroke with percutaneous coronary intervention compared with on-pump and off-pump coronary artery bypass graft surgery: Evidence from a comprehensive network meta-analysis. Am Heart J. 2013;165:910–7.e14. 10.1016/j.ahj.2013.03.011 23708161

[24] Smit Y, Vlayen J, Koppenaal H, Eefting F, Kappetein AP, Mariani MA. Percutaneous coronary invervention versus coronary artery bypass grafting: a meta-analysis. J Thorac Cardiovasc Surg. 2015;149:831-8.e1-13. 10.1016/j.jtcvs.2014.10.112 25467373

[25] Mao Z, Zhong X, Yin J, Zhao Z, Hu X, Hackett ML. Predictors associated with stroke after coronary artery bypass grafting: a systematic review. J Neurol Sci. 2015;357:1–7. 10.1016/j.jns.2015.07.006 26208801

[26] Calvet D, Mas J-L. Recent advances in carotid angioplasty and stenting. Int J Stroke. 2016;11:19–27. 10.1177/1747493015616637 26763017

[27] Ratilal BO, Arroja MMC, Rocha JPF, Fernandes AMA, Barateiro AP, Brites DMTO, et al. Neuroprotective effects of erythropoietin pretreatment in a rodent model of transient middle cerebral artery occlusion. J Neurosurg. 2014;121:55–62. 10.3171/2014.2.JNS132197 24702327

[28] Wang L, Zhang Z, Wang Y, Zhang R, Chopp M. Treatment of stroke with erythropoietin enhances neurogenesis and angiogenesis and improves neurological function in rats. Stroke. 2004;35:1732–7. 10.1161/01.STR.0000132196.49028.a4 15178821

[29] Walberer M, Tschernatsch M, Fischer S, Ritschel N, Volk K, Friedrich C, et al. RNase therapy assessed by magnetic resonance imaging reduces cerebral edema and infarction size in acute stroke. Curr Neurovasc Res. 2009;6:12–9. 10.2174/156720209787466037 19355922

[30] Hamm RJ, Pike BR, O’Dell DM, Lyeth BG, Jenkins LW. The rotarod test: an evaluation of its effectiveness in assessing motor deficits following traumatic brain injury. J Neurotrauma. 1994;11:187–96. 10.1089/neu.1994.11.187 7932797

[31] Obert M, Schulte-Geers C, Schilling RL, Harth S, Kläver M, Traupe H, et al. High-resolution flat-panel volumetric CT images show no correlation between human age and sagittal suture obliteration – independent of sex. Forensic Sci Int. 2010;200:180.e1-12. 10.1016/j.forsciint.2010.04.006 20471762

[32] Juenemann M, Goegel S, Obert M, Schleicher N, Ritschel N, Doenges S, et al. Flat-panel volumetric computed tomography in cerebral perfusion: evaluation of three rat stroke models. J Neurosci Methods. 2013;219:113–23. 10.1016/j.jneumeth.2013.07.010 23880321

[33] Gerriets T, Stolz E, Walberer M, Müller C, Kluge Al, Kaps M, et al. Middle cerebral artery occlusion during MR-imaging: investigation of the hyperacute phase of stroke using a new in-bore occlusion model in rats. Brain Res Brain Res Protoc. 2004;12:137–43. 10.1016/j.brainresprot.2003.08.006 15013464

[34] Vahedi K, Hofmeijer J, Juettler E, Vicaut E, George B, Algra A, et al. Early decompressive surgery in malignant infarction of the middle cerebral artery: a pooled analysis of three randomised controlled trials. Lancet Neurol. 2007;6:215–22. 10.1016/S1474-4422(07)70036-4 17303527

[35] Schwab S, Steiner T, Aschoff A, Schwarz S, Steiner HH, Jansen O, et al. Early hemicraniectomy in patients with complete middle cerebral artery infarction. Stroke. 1998;29:1888–93. 10.1161/01.str.29.9.1888 9731614

[36] Engelhorn T, von Kummer R, Reith W, Forsting M, Doerfler A. What is effective in malignant middle cerebral artery infarction: reperfusion, craniectomy, or both? An experimental study in rats. Stroke. 2002;33:617–22. 10.1161/hs0202.102374 11823679

[37] Hofmeijer J, Schepers J, Veldhuis WB, Nicolay K, Kappelle LJ, Bär PR, et al. Delayed decompressive surgery increases apparent diffusion coefficient and improves peri-infarct perfusion in rats with space-occupying cerebral infarction. Stroke. 2004;35:1476–81. 10.1161/01.STR.0000128415.31274.3a 15131314

[38] Bernaudin M, Marti HH, Roussel S, Divoux D, Nouvelot A, MacKenzie ET, et al. A potential role for erythropoietin in focal permanent cerebral ischemia in mice. J Cereb Blood Flow Metab. 1999;19:643–51. 10.1097/00004647-199906000-00007 10366194

[39] Dahlberg SA, Xu L, Hess DC, Hohnadel E, Hill WD, Fagan SC. Erythropoietin and Erythropoietin Mimetic Peptide in Focal Cerebral Ischemia. Stroke. 2004;35:279.

[40] Wang R, Wu X, Liang J, Qi Z, Liu X, Min L, et al. Intra-artery infusion of recombinant human erythropoietin reduces blood-brain barrier disruption in rats following cerebral ischemia and reperfusion. Int J Neurosci. 2015;125:693–702. 10.3109/00207454.2014.966354 25226558

[41] Chi OZ, Hunter C, Liu X, Weiss HR. Effects of erythropoietin on blood-brain barrier disruption in focal cerebral ischemia. Pharmacology. 2008;82:38–42. 10.1159/000127839 18434762

[42] Zechariah A, ElAli A, Hermann DM. Combination of tissue-plasminogen activator with erythropoietin induces blood-brain barrier permeability, extracellular matrix disaggregation, and DNA fragmentation after focal cerebral ischemia in mice. Stroke. 2010;41:1008–12. 10.1161/STROKEAHA.109.574418 20360548

[43] Li Y, Lu Z-Y, Ogle M, Wei L. Erythropoietin prevents blood brain barrier damage induced by focal cerebral ischemia in mice. Neurochem Res. 2007;32:2132–41. 10.1007/s11064-007-9387-9 17562165

[44] Gunnarson E, Song Y, Kowalewski JM, Brismar H, Brines M, Cerami A, et al. Erythropoietin modulation of astrocyte water permeability as a component of neuroprotection. Proc Natl Acad Sci USA. 2009;106:1602–7. 10.1073/pnas.0812708106 PMC2629445 19164545

[45] Calapai G, Marciano MC, Corica F, Allegra A, Parisi A, Frisina N, et al. Erythropoietin protects against brain ischemic injury by inhibition of nitric oxide formation. Eur J Pharmacol. 2000;401:349–56. 10.1016/s0014-2999(00)00466-0 10936493

[46] Blasig IE, Mertsch K, Haseloff RF. Nitronyl nitroxides, a novel group of protective agents against oxidative stress in endothelial cells forming the blood-brain barrier. Neuropharmacology. 2002;43:1006–14. 10.1016/s0028-3908(02)00180-6 12423670

[47] Bahcekapili N, Uzüm G, Gökkusu C, Kuru A, Ziylan Y. The relationship between erythropoietin pretreatment with blood-brain barrier and lipid peroxidation after ischemia/reperfusion in rats. Life Sci. 2007;80:1245–51. 10.1016/j.lfs.2006.12.014 17300815

[48] Utepbergenov DI, Mertsch K, Sporbert A, Tenz K, Paul M, Haseloff RF, et al. Nitric oxide protects blood-brain barrier in vitro from hypoxia/reoxygenation-mediated injury. FEBS Lett. 1998;424:197–201. 10.1016/s0014-5793(98)00173-2 9539150

[49] Xiong Y, Mahmood A, Qu C, Kazmi H, Zhang ZG, Noguchi CT, et al. Erythropoietin improves histological and functional outcomes after traumatic brain injury in mice in the absence of the neural erythropoietin receptor. J Neurotrauma. 2010;27:205–15. 10.1089/neu.2009.1001 PMC2824224 19715391

[50] Li Y, Lu Z, Keogh CL, Yu SP, Wei L. Erythropoietin-induced neurovascular protection, angiogenesis, and cerebral blood flow restoration after focal ischemia in mice. J Cereb Blood Flow Metab. 2007;27:1043–54. 10.1038/sj.jcbfm.9600417 17077815

[51] Murphy AM, Xenocostas A, Pakkiri P, Lee T-Y. Hemodynamic effects of recombinant human erythropoietin on the central nervous system after subarachnoid hemorrhage: reduction of microcirculatory impairment and functional deficits in a rabbit model. J Neurosurg. 2008;109:1155–64. 10.3171/JNS.2008.109.12.1155 19035736

[52] Shafi NI, Andresen J, Marrelli SP, Bryan RM. Erythropoietin potentiates EDHF-mediated dilations in rat middle cerebral arteries. J Neurotrauma. 2008;25:257–65. 10.1089/neu.2007.0347 18352839

[53] Zhang F, Xing J, Liou AK-F, Wang S, Gan Yu, Luo Y, et al. Enhanced delivery of erythropoietin across the blood-brain barrier for neuroprotection against ischemic neuronal injury. Transl Stroke Res. 2010;1:113–21. 10.1007/s12975-010-0019-3 PMC2888513 20577577

[54] Leist M, Ghezzi P, Grasso G, Bianchi R, Villa P, Fratelli M, et al. Derivatives of erythropoietin that are tissue protective but not erythropoietic. Science. 2004;305:239–42. 10.1126/science.1098313 15247477

